# Ocular Histopathological Findings in Semi-Domesticated Eurasian Tundra Reindeer (*Rangifer tarandus tarandus*) with Infectious Keratoconjunctivitis after Experimental Inoculation with Cervid Herpesvirus 2

**DOI:** 10.3390/v12091007

**Published:** 2020-09-09

**Authors:** Javier Sánchez Romano, Karen K. Sørensen, Anett K. Larsen, Torill Mørk, Morten Tryland

**Affiliations:** 1 Arctic Infection Biology Research Group, Department of Arctic and Marine Biology, Faculty of Biosciences, Fisheries and Economics, UiT The Arctic University of Norway, 9019 Tromsø, Norway; 2 Vascular Biology Research Group, Department of Medical Biology, Faculty of Health Sciences, UiT The Arctic University of Norway, 9019 Tromsø, Norway; karen.sorensen@uit.no (K.K.S.); anett.k.larsen@uit.no (A.K.L.); 3 Section for Research in Food Safety and Animal Health, Norwegian Veterinary Institute, 9010 Tromsø, Norway; torill.mork@vetinst.no

**Keywords:** alpha-herpesvirus, CvHV2, histology, infectious keratoconjunctivitis, ophthalmology, pathology, reindeer, wildlife

## Abstract

Infectious keratoconjunctivitis (IKC) is a common transmissible ocular disease in semi-domesticated Eurasian tundra reindeer (*Rangifer tarandus tarandus*). In large outbreaks, IKC may affect tens of animals in a herd, with the most severe cases often requiring euthanasia due to the destruction of the affected eyes and permanent blindness. An experimental inoculation with cervid herpesvirus 2 (CvHV2), alone or in combination with *Moraxella bovoculi*, demonstrated that CvHV2 has the ability to cause clinical signs of IKC in previously unexposed reindeer. Tissues collected from upper and lower eyelids, lacrimal gland and cornea, were processed for light and transmission electron microscopy. Histopathological analysis of the eyes inoculated with CvHV2 showed widespread and severe pathological findings. Mucosal tissues from these eyes showed fibrinous and purulent exudates, hyperemia, hemorrhages, necrosis, vascular thrombosis, vascular necrosis, infiltration of mononuclear cells and neutrophils, and lymphoid follicle reaction, which matches the described histopathology of IKC in reindeer. Characteristic alpha-herpesvirus particles matching the size and morphology of CvHV2 were identified by transmission electron microscopy in the conjunctival tissue. The quantification of viral particles by qPCR revealed high copy numbers of viral DNA in all CvHV2 inoculated eyes, but also in the non-inoculated eyes of the same animals. The histopathology of eye tissues obtained from the CvHV2 inoculated reindeer and the lack of inflammation from bacterial infection, together with the detection of CvHV2 DNA in swabs from the inoculated and non-inoculated eyes of the same animals, verified that CvHV2 was the primary cause of the observed histopathological changes.

## 1. Introduction

Infectious keratoconjunctivitis (IKC) is among the most common transmissible ocular diseases in ruminants worldwide. Clinical cases are reported in numerous domestic and wild ruminant species and have been associated with a great variety of bacterial and viral agents as well as environmental stress factors [1,2,3,4,5,6,7,8,9,10]. IKC has been reported from herds of semi-domesticated Eurasian tundra reindeer (*Rangifer tarandus tarandus*) in Fennoscandia for more than 100 years [11] and is nowadays a rather common disease [12], appearing as individual cases or as outbreaks, affecting many animals [13,14]. IKC in reindeer has been considered a multifactorial eye disease, but cervid herpesvirus 2 (CvHV2) and *Moraxella bovoculi* were recently suggested as possible primary causative agents, with both pathogens being isolated during clinical outbreaks of the disease [14,15]. Furthermore, the presence and quantity of CvHV2 was found positively associated with the severity of clinical symptoms during the early stages of the disease during an outbreak in Norway [14], and was also found to be significantly associated with the presence of clinical symptomatology in a previous study, addressing potential causative pathogens for IKC [15].

CvHV2 is a member of the genus *Varicellovirus*, in the subfamily *Alphaherpesvirinae* of the *Herpesviridae* family [16], and is endemic in reindeer in Norway, Sweden and Finland [17,18,19].

In a recent controlled challenge experiment, we found that CvHV2 causes IKC in previously unexposed and seronegative semi-domesticated reindeer [20]. Animals inoculated with CvHV2, alone or in combination with the gram-negative bacterium *M. bovoculi*, developed clinical signs of IKC within two days after inoculation of the virus, and displayed conjunctival edema, hyperemia, and purulent exudate. The severity of the clinical signs increased with time during the experimental period (up to euthanasia at two–seven days post inoculation), leading to ulcer-like lesions in cornea in three animals (later found to be deep erosions, 3–12 mm wide, but not ulcers, in the histopathological examination (this paper)). The animals were euthanized according to protocol as soon as sign of corneal ulcers over 6 mm in diameter appeared. *Moraxella bovoculi* did not produce clinical signs in this controlled challenge experiment, except for a transient grey or whitish ocular discharge on the day of inoculation. In the present paper, we present the detailed histopathology of eye tissues collected from the previous challenge experiment [20].

There are few descriptions of the histopathology of IKC in reindeer. Early studies [13,21,22], which claimed the multifactorial nature of the disease, described a purulent/necrotizing conjunctivitis. In cases with keratitis, there were corneal edema and infiltration of leukocytes/neutrophils in the corneal stroma [13,21,22]. Similar pathological changes were described in conjunctiva of semi-domesticated reindeer with purulent conjunctivitis in an outbreak of ulcerative and necrotizing stomatitis and rhinitis caused by a herpesvirus closely related to bovine herpesvirus 1 [23]. A similar virus had previously been isolated and characterized by Ek-Kommonen et al. [24]. In 2009, CvHV2 was reported as the primary agent of a clinical outbreak of IKC in semi-domesticated reindeer in Troms County, Norway, where samples from 28 animals with clinical symptoms, and 12 healthy animals, were investigated for the presence of CvHV2 (by virus isolation and PCR), antibodies against CvHV2, and bacterial cultivation [14]. The eyes of three animals that were euthanized during the outbreak due to severe IKC were subjected to histology. Each animal showed affection of a single eye only. In two of the animals, the affected eyes were totally collapsed and destroyed whereas in the third animal, the affected eye was still intact but showed corneal edema and ulceration. Degeneration and necrosis of epithelial cells of the conjunctiva and lacrimal glands were also seen [14].

The aim of the present study was to investigate the pathogenesis of IKC in reindeer caused by CvHV2, by characterizing early histopathological changes in various eye tissues of reindeer that were experimentally inoculated with CvHV2 and that developed IKC [20]. We also adapted a quantitative PCR assay to be able to quantify virus in the conjunctiva of the CvHV2-inoculated eyes and the non-inoculated eyes during the experimental period.

## 2. Materials and Methods 

### 2.1. Ethics

Tissues and samples were collected from reindeer included in a previously approved animal experiment (Norwegian Animal Research Authority; ID 5880) [20]. The experiment aimed at identifying the transmissible and causative agent of IKC in semi-domesticated reindeer to better understand the pathogenesis and to improve prophylaxis and medical treatment during disease outbreaks.

### 2.2. Collection and Processing of Eye Tissues for Histological Examination

Tissues were collected from both eyes of 18 one-year-old reindeer calves within minutes after euthanasia, as part of a study of experimentally induced IKC [20]. In brief, the animals, which were all seronegative for alpha-herpesvirus and culture negative for *M. bovoculi* (eye swab samples) at the start of the experiment, were sedated (medetomidine/ketamine). Local anesthetic (oxibuprokain) was applied to the right eye of all experimental animals, including negative control animals. The conjunctival mucosa was then mildly rubbed with sterile sandpaper (grade 60) and inoculated with CvHV2 (group 1, n = 5), CvHV2 and *M. bovoculi* (group 2, n = 5), *M. bovoculi* (group 3, n = 5), or sterile saline alone (group 4, n = 3, negative control) (Table 1). CvHV2 was inoculated by introducing a sterile cotton pad soaked in sterile phosphate-buffered saline (PBS) with CvHV2 (5.8 × 10^6^ TCID_50_/ml) under the lower eyelid and keeping it there for 4 min. *Moraxella bovoculi* was inoculated onto the conjunctiva using a cotton swab that was skimmed over an agar plate with pure bacterial culture [20]. Control animals were inoculated in the same way, but with sterile saline only. Both the viral and the bacterial isolates used for inoculation had been obtained from reindeer with clinical signs of IKC. All animals inoculated with CvHV2 (groups 1 and 2) developed clinical signs of IKC within two–three days, whereas animals inoculated with *M. bovoculi* alone or sterile saline did not develop IKC during the study period of 15 days [20].

Samples for electron microscopy (EM) were collected from the medial angle of the upper and lower eyelids immediately after euthanasia. The tissue samples were cut in 1 mm^3^ pieces, fixed in McDowell Trump’s fixative (4.0 % formaldehyde and 1.0 % glutaraldehyde in phosphate buffer, pH 7.2) [25], postfixed in 1.0 % osmium tetroxide in water, dehydrated through a graded ethanol series (30-100 %) and embedded in epon for ultramicrotomy. Semi-thin sections (500 nm) were stained with toluidine blue to localize areas of interest for the TEM analysis. Ultrathin sections (70 nm) were obtained using a Leica EM UC6 ultramicrotome (Leica Microsystems, Vienna, Austria) with a Diatome diamond knife (Diatome, Biel, Switzerland) and stained with 1.0 % uranyl acetate and Reynolds lead citrate and examined with a Jeol JEM 1010 transmission electron microscope (Jeol USA Inc., Peabody, MA, USA) connected to a Morada Camera system (Olympus Soft Imaging Solutions, Münster, Germany).

For light microscopy, the eye globe with conjunctiva and eyelids were dissected out and fixed in 4.0 % phosphate buffered formaldehyde, pH 7.2. After proper fixation (> 1 week), samples from cornea, the upper and lower eyelids, the third eyelid and the lacrimal gland were collected from each eye and processed routinely for paraffin embedding. Sections of 5 μm were stained with hematoxylin and eosin (HE) and encoded with a four digits code for blind histological examination. A form was created for each tissue for standardization of the histopathological evaluation between the different researchers. Histopathological changes in the eye tissues examined were graded on a five-grade severity scale, with a score for each major structure in the tissue (Table 1). In the upper eyelid, the skin, Meibomian gland and the conjunctival mucosa were evaluated. The evaluation was similar for the lower eyelid, with the addition of third eyelid and the accessorial lacrimal gland, if present. In sections of cornea, the limbus, corneal epithelium, and stroma were evaluated. In sections of the lacrimal gland, pathological changes in the glandular epithelium, excretory ducts and interstitium were described and graded. For each tissue, we registered the presence of hyperemia, edema, exudates, hemorrhages, vascular thrombosis, tissue necrosis, inflammatory cell infiltration, and lymphoid follicle reaction or necrosis. We further included an overall score of the pathological findings in the tissues examined, with 1 being normal tissue, and 5 being generalized severe inflammation and/or necrosis (Table 1).

Tissue sections from all lower eyelids and lacrimal glands were also stained with a modified Gram-staining technique [26] and evaluated for the presence of bacteria.

### 2.3. Quantitative Real-Time PCR (qPCR)

An assay for quantification of virus particles in the eyes of the animals inoculated with CvHV2 (groups 1 and 2) was established. DNA was isolated from conjunctival swab samples available from the challenge experiment [20] using Maxwell^®^ 16 LEV Buccal swab DNA kit (Promega, WI, USA) following the manufacturer’s instructions with slight modifications. In summary, 300 μl of the swab storage medium was mixed with 30 μl Proteinase K and 300 μl Lysis buffer. This solution was incubated at 56 °C for 20 min. After the incubation, 350 μl of the lysed mixture was added to well #1 of the Maxwell^®^ 16 LEV cartridge, which was then processed as indicated by the manufacturer, with a final elution volume of 100 μl. A quantitative real-time Taqman Probe based PCR (qPCR) for the detection of herpesvirus DNA was performed as described previously [27] with slight modifications [20]. Samples were run in triplicate, including a standard curve for quantification of the viral DNA copies.

For the standard curve, CvHV2 inoculum amplicons produced by qPCR were cloned into competent *Escherichia coli* cells with the TOPO^®^ TA^®^ cloning kit for sequencing following the manufacturer’s instructions (Invitrogen, Karlsruhe, Germany). To generate the standard curve for qPCR, bacterial DNA from the clones was extracted and DNA concentrations were measured with a Qubit 2.0 fluorometer (Invitrogen, Life Technologies, USA). Extracted DNA was diluted in order to have 10^7^ DNA copies/μl from each clone, and serial ten-fold dilutions were prepared and tested by qPCR. Serial dilutions for the standard curve were stored at −20 °C until further use.

## 3. Results

### 3.1. Histopathology

**Figure 1:**
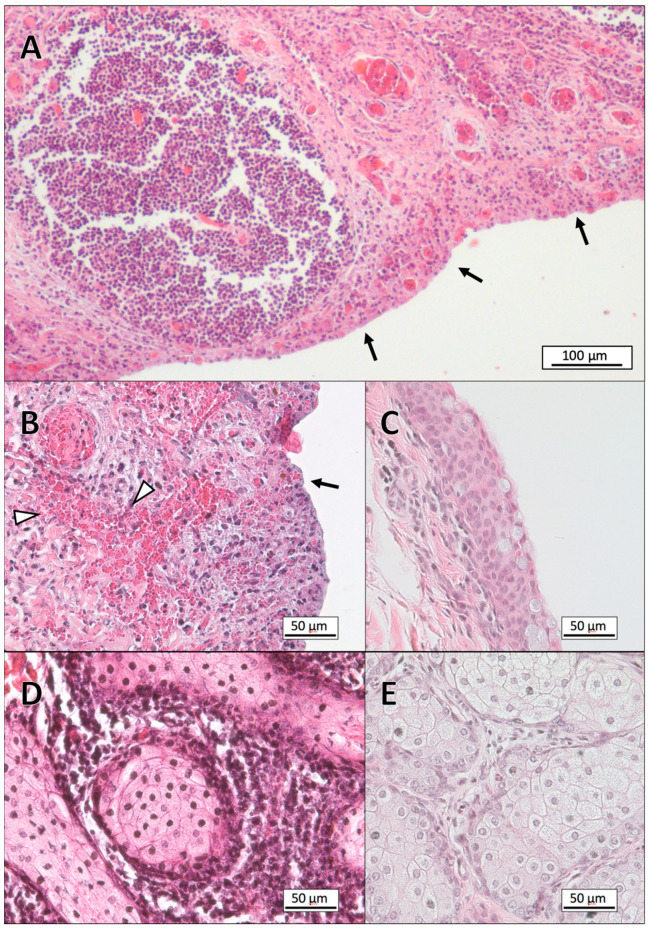
Micrographs of paraffin sections of the lower eyelid of Eurasian tundra reindeer inoculated with CvHV2 (**A,B,D**), and of a control animal (**C,E**). The tissue sections were stained with hematoxylin and eosin (HE). (**A**) Overview image of conjunctival tissue two days after inoculation with CvHV2 (group 1), showing total loss of the epithelial layer (arrows), and acute inflammation in the lamina propria. (**B**) Higher magnification of conjunctival tissue from the same animal, showing severe inflammation and hemorrhage (arrow heads). The arrow is pointing to the conjunctival surface. (**C**) Conjunctiva of a control animal. (**D**) Meibomian gland from a reindeer inoculated with both CvHV2 and *Moraxella bovoculi* (group 2), showing inflammation with infiltration of mostly mononuclear cells. (**E**)Meibomian gland of a control animal.

**Figure 2:**
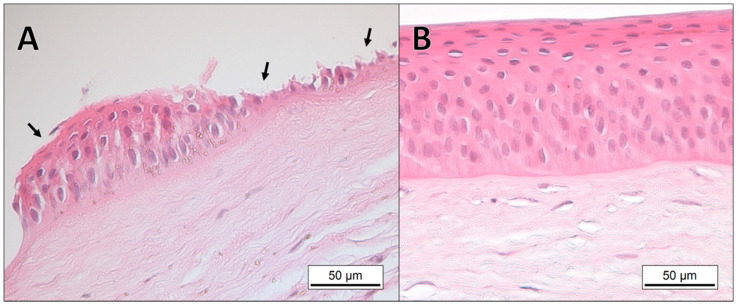
Micrographs of paraffin sections of corneal tissue of a reindeer euthanized five days after inoculation with CvHV2 and *Moraxella bovoculi* (**A**), and a control animal (**B**). The tissue sections are stained with hematoxylin and eosin (HE). Arrows in (**A**) are pointing at erosions of the corneal epithelium. Basal membrane was still intact.

**Figure 3:**
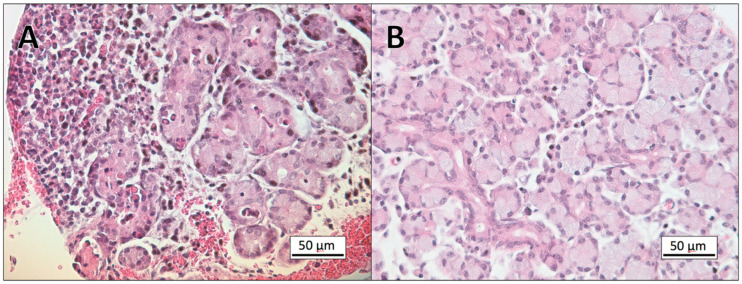
Micrographs of paraffin sections of the lacrimal gland of a reindeer euthanized six days post inoculation with CvHV2 (**A**), and a control animal (**B**). The tissue sections are stained with hematoxylin and eosin (HE). The image in (**A**) shows acute inflammation with destruction of glandular tissue, hyperemia, hemorrhage and accumulation of inflammatory cells, dominated by neutrophils.

The result of the histological evaluation of eye tissues from the 18 reindeer included in the study are summarized in Table 1. Histological examination of the tissues collected from the experimental animals inoculated with CvHV2, alone (group 1) or in combination with *M. bovoculi* (group 2) showed epithelial necrosis and acute inflammation in all tissues from the inoculated eyes. The most severe lesions were already present in the upper and lower eyelids, with scores of 4 to 5 in all examined tissues, from 2 days post-inoculation (p.i). The conjunctival epithelium was completely eroded, and the sub-epithelial connective tissue showed focal or generalized severe inflammation, with hyperemia, fibrinous exudation, infiltration of neutrophils and mononuclear leukocytes, and hyperplasia and/or lymphoid follicle necrosis. Severe and widespread vascular pathology was also present, with vasculitis and vascular necrosis leading to hemorrhages (Figure 1). The pathological severity in the skin of the eyelids and in Meibomian glands increased through the experiment, with focal areas of necrosis in the epidermis and dermis and infiltration of mononuclear leukocytes in the dermis and in the interstitium of the Meibomian glands as the most prominent signs (Figure 1). At day 2 p.i., mild corneal edema and focal erosions in the conjunctiva of limbus were seen, progressing to erosions in the corneal epithelium, and focal necrosis and leukocyte infiltration in limbus, in eyes of animals euthanized on days 4 to 7 p.i. (Figure 2). Lacrimal glands showed no apparent pathological findings in CvHV2 inoculated animals that were euthanized during the first three days. In animals euthanized at day 4–7, focal glandular necrosis and moderate to severe inflammation of excretory ducts and glandular tissues were observed, with infiltration of neutrophils and mononuclear leukocytes in ducts, glandular lumen, and interstitium (Figure 3).

No pathological findings were identified in the conjunctiva or cornea of the samples collected from the inoculated eyes of animals in group 3 (inoculated with *M. bovoculi* alone) or group 4 (controls) (Figure 1, Figure 2 and Figure 3). In all groups, the samples from the non-inoculated eyes (i.e. left eye) showed normal histology.

### 3.2. Gram-Staining of Tissue Sections

Sections from the lower eyelid and lacrimal glands of inoculated eyes in all experimental groups (Table 1) were stained with a modified Gram technique [26] in order to identify bacterial infection. A few, scattered groups of gram-positive and gram-negative bacteria were observed in histological slides from the lower eyelids from a few animals. The bacteria were located at the surface of the tissue samples.

### 3.3. Electron Microscopy

**Figure 4:**
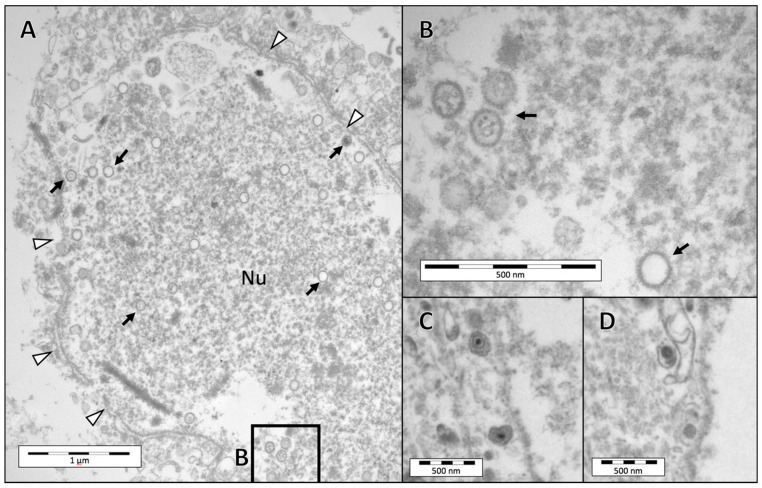
Transmission electron micrographs of the conjunctival epithelium of the lower eyelid of a reindeer (R17) euthanized three days after inoculation with both CvHV2 and *M. bovoculi*. (**A**) Cell nucleus (Nu; nuclear membrane demarcated by arrow heads) of a necrotic cell close to the conjunctival surface, containing assembled CvHV2 nucleocapsids (arrows). (**B**) High magnification image of insert in A showing details of viral nucleocapsids. (**C,D**) Enveloped virus particles detected close to the conjunctival surface (arrows).

**Figure 5:**
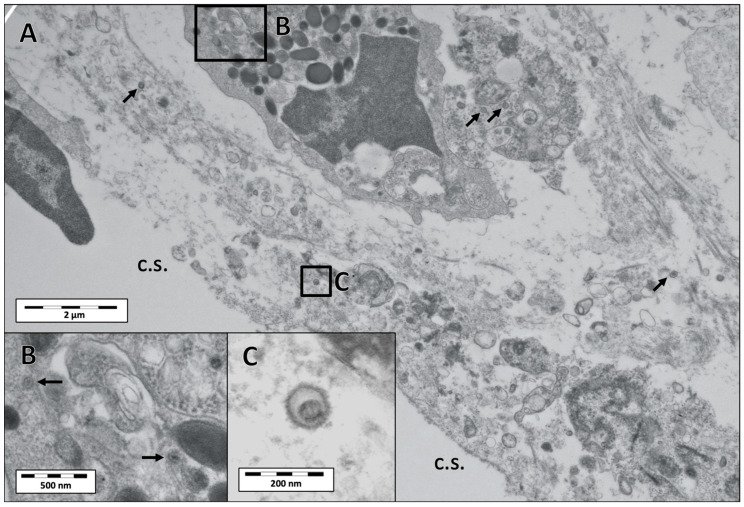
Transmission electron micrographs of the conjunctiva of the lower eyelid of a reindeer (R17) euthanized three days after inoculation with both CvHV2 and *Moraxella bovoculi* (R17). (**A**) The image is taken from an area close to the conjunctival surface (c.s.) and shows cellular necrosis and a neutrophil. Arrows in (**A**) are pointing to viral capsids in the necrotic tissue. (**B**) Higher magnification of the insert (**B**) in the neutrophil in (**A**) showing two viral capsids in the cytosol of this cell (arrows). (**C**) Higher magnification of the viral capsid in insert (**C**) in image (**A**).

Electron microscopy was conducted on conjunctival tissue from the lower eyelid of a reindeer that was inoculated with CvHV2 and *M. bovoculi* (group 2) and euthanized at day 2 [20]. The examination revealed pronounced epithelial necrosis. Herpes viral capsids were detected in cell nuclei of necrotic cells (Figure 4), and in the cytoplasm of a neutrophil (Figure 5). Enveloped viral particles were detected in the surface of the necrotic conjunctival tissue (Figure 4), indicating viral replication. The ultrastructure and size of the virus particles corresponded to previous descriptions and images of CvHV2 [23,28].

### 3.4. Quantitative Real-Time PCR (qPCR)

**Figure 6:**
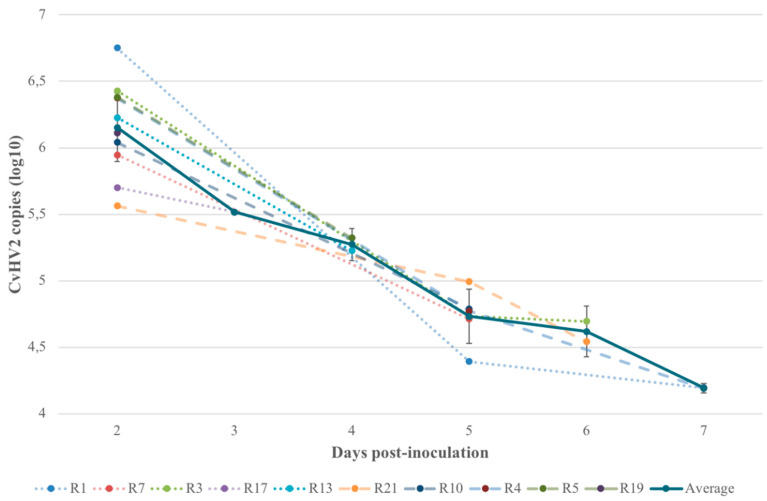
The number of viral copies, shown as log10 of the quantification data, identified by qPCR on eye swab samples from the inoculated (right) eyes of all reindeer inoculated with CvHV2 (alone or in combination with *Moraxella bovoculi*; groups 1 and 2, respectively) from day 2 post inoculation until euthanasia.

Results from the quantification of viral particles in swab samples collected from the inoculated right eye of the animals challenged with CvHV2 (groups 1 and 2) showed a similar pattern in all animals, with a 100-fold reduction in average virus load from day 2 p.i. (1.89 x 10^6^; C_t_ = 19.47) until day 7 p.i. (1.55 x 10^4^; C_t_ = 25.68) (Figure 6). A maximum of 5.64 x 10^6^ copies (C_t_ = 17.55) (Figure 6) were detected on day 2 p.i. (R1).

**Figure 7:**
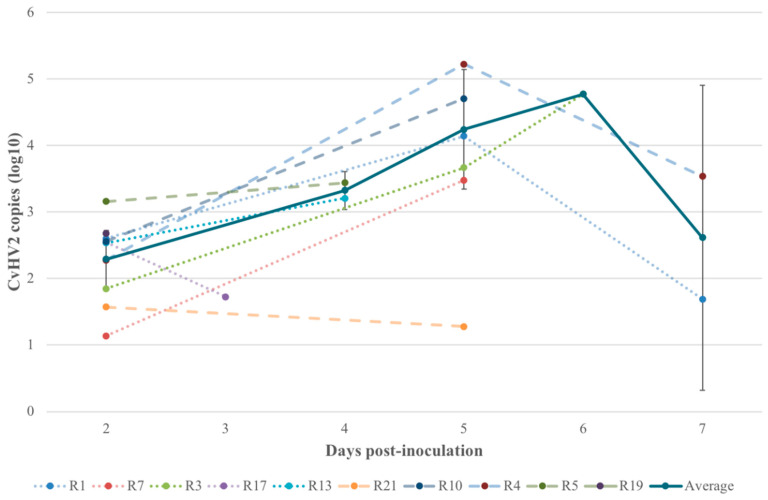
The number of viral copies, shown as log10 of the quantification data, identified by qPCR on eye swab samples from the non-inoculated (left) eyes of all reindeer inoculated with CvHV2 (alone or in combination with *Moraxella bovoculi*; groups 1 and 2, respectively) from day 2 post inoculation until euthanasia. Animals R17 and R21 were defined as outliers and were excluded from the average and standard error calculations.

In contrast, we observed an increase in CvHV2 copies in the non-inoculated left eye of the infected animals, with 3.68 x 10^2^ detected copies (C_t_ = 32.36) in average on day 2 p.i. and up to 5.89 x 10^4^ copies (C_t_ = 24.1) on day 6 p.i. (R3). This trend changed on day 7 p.i., with the last euthanized reindeer, R1 (group 1) and R4 (group 2), showing a decrease in the CvHV2 copies from the previous sampling date (Figure 7). The exceptions to this pattern were animals R17 and R21, from which no peak was evident after the initial sampling point – the two animals showed a decrease in the number of CvHV2 copies in the left eye throughout the experiment (Figure 7).

## 4. Discussion

During clinical outbreaks of IKC that receive veterinary attention, animals are often investigated and sampled when the disease has already affected several animals. Thus, samples will be from animals with IKC at different stages, and without knowledge of the onset, the time point of infection of each animal, the infective load, the port of entry of the pathogen, or the potential contribution of secondary infections, which makes it difficult or impossible to reveal the primary agent. Controlled experimental inoculation studies are thus necessary tools in veterinary medicine to be able to reveal further knowledge on the infection biology parameters.

The histopathology revealed that CvHV2 infection caused severe pathology (i.e. inflammation, necrosis) in all the ocular structures investigated in this study, including conjunctival tissue, cornea, lacrimal glands, and skin of the eyelids. Furthermore, the pathological findings were consistent with the clinical signs and macroscopic lesions observed in this controlled challenge experiment. Clinical signs were observed between days 1 and 2 p.i. [20] and conjunctival epithelial necrosis and corneal erosion were already observed in the first animal that was euthanized, on day 2.

The histopathology of the eye tissues of the animals that had been inoculated with CvHV2 further matched with previous descriptions of IKC pathological findings in semi-domesticated reindeer [13,14,21,22,23]. The presence of herpesvirus particles, including empty capsids in cell nuclei and enveloped viral particles in the conjunctival tissues, were also observed by TEM (Figure 4 and Figure 5), confirming viral replication in the host conjunctival cells. The absence of histopathological changes in tissues obtained from the *M. bovoculi* and non-inoculated control groups (groups 3 and 4, respectively), together with the negative results from those animals in the CvHV2 qPCR performed by Tryland et al. [20], further supports our conclusion that CvHV2 caused the described pathology. Furthermore, CvHV2 quantification by qPCR in this study revealed high yield of CvHV2 DNA in the inoculated eyes of all animals in the CvHV2 inoculated groups (Table 1 and Figure 6). Altogether, these results are supporting the theory of CvHV2 as a primary causative agent of IKC in semi-domesticated reindeer [14,15,20].

Among the animals inoculated with CvHV2 (groups 1 and 2), the lower right eyelid, which was the port of inoculation, showed the most severe histopathology, with an average score of 4.7 out of 5. Destruction of the epithelium of the conjunctiva and severe inflammation in underlying tissues were also observed in the upper eyelids, with an average score of 4.2, whereas the corneal lesions varied from mild focal erosions (score 2) to severe erosion (score 4), with an average score of 3.4. The lowest average score was recorded in lacrimal glands, with 2.5 out of 5. The lacrimal glands showed no apparent pathology in the animals euthanized early in the experiment (days 2–4 p.i.). Lacrimal glands are located in the upper lateral region of the orbits and lay deep in the periorbital tissue and most distant from the port of inoculation. This implies that the CvHV2 infection of the glands was not due to contamination or vectors, but a progression of the disease to deeper tissues.

In a previous experimental infection with CvHV2 in reindeer, where the animals were inoculated intratracheally or intravaginally [29], immunohistochemistry for CvHV2 of lung and uterine tissues showed strong positive staining of goblet cells of the respiratory epithelium and basal cells of endometrial glands. Positive virus staining was also observed in leukocytes. This virus tropism is consistent with the severe epithelial and glandular damage described in the eye tissues in the present study, and with our observation of enveloped viral particles and viral capsids in the necrotic eye tissue, and viral capsids inside a neutrophil, by electron microscopy (Figure 5). Unfortunately, the monoclonal antibodies used in the study by das Neves et al. [29] are no longer available.

The histopathological findings and qPCR results in the present study explain the quick development of clinical signs in the animals described in [20]. The number of detected CvHV2 DNA copies in the inoculated eyes, as indicated by the qPCR results, decreased in the CvHV2 inoculated eyes throughout the experimental period (up to 7 days) (Figure 6). An explanation for this may be that the conjunctival epithelium was eroded within the first 24 hours p.i. with the amount of virus decreasing as the available replication sites disappeared due to tissue destruction.

In all animals inoculated with CvHV2, the non-inoculated left eye was also positive for the presence of CvHV2 DNA (Table 1 and Figure 7). The viral load was low compared to the inoculated eye but showed an increase in the amount of virus in most animals until day 6 p.i., followed by a decrease at day 7 (end of the experiment) (Figure 7). The decrease at day 7 matched with the first day of detection of antibodies against CvHV2 in the same animals [20], suggesting an active immune response to the infection. In accordance with this observation, one animal (R21) that showed an atypical decrease in the number of viral particles detected in days 2, 5 and 6 p.i. (this study) had antibodies against CvHV2 above threshold detection level already at day 6 p.i. [20]. This suggests that this animal had an early immune response to the infection, thus being able to slow down the replication of CvHV2 in the non-inoculated eye. The presence of viral DNA in the non-inoculated eye of the animals in groups 1 and 2, indicates that CvHV2 can be easily transferred from the point of inoculation to other tissues. Even though viremia was not detected in the CvHV2 inoculated animals [20], spreading may occur by direct contact with secretions from the inoculated eye, transmission through the lacrimal ducts to the nasal cavity, or through mechanical transfer by insect vectors.

A low number of gram-positive and gram-negative bacteria was detected in the histological slides of the lower eyelids from some of the inoculated eyes independently of the experimental group. The bacteria were situated on the surface of the tissue and considered without pathological relevance. During the experiment [20], *M. bovoculi* was isolated from eye swabs from 8 of 10 of the reindeer inoculated with the bacteria. In addition, poor growth of a few colonies of *M. bovoculi* in mixed cultures from eye swabs from four of five animals inoculated only with CvHV2 was described. This growth was considered a contamination from the environment. Of note, animals inoculated with *M. bovoculi* alone did not show symptoms of IKC [20], which was confirmed by the histological examination of eye samples from the same animals (this study). The *M. bovoculi* strain used in this experiment had been isolated from semi-domesticated reindeer with IKC during an outbreak but was not specifically tested for virulence factors. Genomic investigations of *M. bovoculi* isolates obtained from the eyes of cattle with infectious bovine keratoconjunctivitis (IBK) and from the nasopharynx of cattle without such clinical signs described large differences in genome structure and the presence of virulence genes between isolates, creating two distinct phylogenetic groups and suggesting the existence of pathogenic and non-pathogenic bacterial strains [30]. Further studies have also indicated a high degree of recombination and genetic diversity between pathogenic strains [31]. The lack of histopathological findings associated with the presence of *M. bovoculi*, the scattered presence of bacteria in the Gram staining, and the studies describing large genetic differences among *Moraxella* strains [30,31], strongly suggest that the bacteria isolated by Tryland et al. [20] may have originated from other locations and not from the original bacterial inoculum, and that they were not the primary cause of pathology.

## 5. Conclusions

We conclude that CvHV2 is able to cause severe histopathology in all ocular structures known to be affected in IKC in reindeer. This is consistent with the clinical signs and macroscopic lesions described previously [20]. The eyes inoculated with CvHV2 (right) quickly developed severe inflammation in conjunctiva and focal corneal erosions. Furthermore, the virus spread to more distant tissues from the port of inoculation, such as the lacrimal glands, causing focal glandular necrosis, and moderate to severe inflammation of excretory ducts and glandular tissues. The virus also spread to the non-inoculated eyes (left) of all the virus-inoculated animals, without visible histopathology but positive detection by qPCR.

## Figures and Tables

**Table 1:** Histopathological evaluation of tissue samples and quantitative real-time PCR (qPCR) results from the inoculated eye of experimentally inoculated semi-domesticated Eurasian tundra reindeer (*Rangifer t. tarandus*). Samples were obtained after euthanasia of animals inoculated with cervid herpesvirus 2 (CvHV2), a combination of CvHV2 and *Moraxella bovoculi*, *M. bovoculi,* or saline (control) in a controlled challenge experiment [20]. N/A: procedure not applied.

Inoculum	ID	General Histopathological Evaluation					Euthanasia	qPCR	
		Upper eyelid ^1^	Lower eyelid ^1^	Cornea ^2^		Lacrimal gland ^1^		Ct ^3^	Viral copies ^3^
**CvHV2 (group 1; n = 5)**	**R19**	N/A	4	2		1	Day 2 p.i.	19.59	1.29 x 10^6^
	R5	4	5	4		4	Day 4 p.i.	22.10	2.10 x 10^5^
	R10	4	5	3		3	Day 5 p.i.	23.78	6.15 x 10^4^
	R21	5	5	3		4	Day 6 p.i.	24.58	3.48 x 10^4^
	R4	5	5	4		2	Day 7 p.i.	25.69	1.55 x 10^4^
**CvHV2 and *M. bovoculi* (group 2; n = 5)**	R17	4	4			1	Day 3 p.i.	21.50	3.28 x 10^5^
	R13	4	5	4		1	Day 4 p.i.	22.37	2.10 x 10^5^
	R7	4	4	4		1	Day 5 p.i.	24.03	5.15 x 10^4^
	R3	4	5	3		4	Day 6 p.i.	24.09	4.96 x 10^4^
	R1	5	5	4		4	Day 7 p.i.	25.67	1.57 x 10^4^
***M. bovoculi* (group 3; n = 5)**	R16	1	1	1		1	Day 3 p.i.	N/A	N/A
	R6	1	1	1		1	Day 6 p.i.	N/A	N/A
	R8	1	1	1		1	Day 13 p.i.	N/A	N/A
	R9	1	1	1		1	Day 13 p.i.	N/A	N/A
	R15	1	1	1		1	Day 13 p.i.	N/A	N/A
**Control (group 4; n = 3)**	R2	1	1	1		1	Day 15 p.i.	N/A	N/A
	R18	1	1	1		1	Day 15 p.i.	N/A	N/A
	R20	1	1	1		1	Day 15 p.i.	N/A	N/A
^1,2^ Histopathological evaluation was graded in a 5-degrees scale system:									
^1^ 1 = No pathological findings, normal tissue 2 = Mild lesions (e.g. mild erosions, lymphoid follicle reaction, no/little inflammatory cell infiltration), 3 = General moderate inflammation 4 = General moderate inflammation with focal severe inflammation 5 = General severe inflammation.					^2^ 1 = No pathological findings, normal tissue, 2 = Mild, focal lesions (e.g. mild erosions, mild edema, mild infiltration of cells at the limbus) 3 = Mild, widespread lesions (e.g. erosions of the corneal epithelium, corneal edema) 4 = Mild, widespread lesions with focal severe erosions with edema and cellular reaction at the corneal lumbus 5 = Corneal ulcer (humane end-point) and severe inflammatory reaction.				
^3^ Viral copies (qPCR) in eye swab samples of the inoculated eye at the day of euthanasia (two–seven days p.i.). Quantification of viral DNA copies was performed in animals inoculated with CvHV2, alone or in combination with *M. bovoculi*. PCR without quantification was previously performed in all experimental reindeer by Tryland et al. [20].									
